# Nutritional Composition and In Vitro Ruminal Digestibility of Crabgrass (*Digitaria sanguinalis* (L.) Scop.) in Monoculture or Interseeded with Cowpea (*Vigna unguiculata* (L.) Walp) and Lablab (*Lablab purpureus* (L.) Sweet)

**DOI:** 10.3390/ani13142305

**Published:** 2023-07-14

**Authors:** Matias Jose Aguerre, Omar Manuel Peña, Cesar Velasquez, Gonzalo Ferreira

**Affiliations:** 1 Department of Animal and Veterinary Sciences, Clemson University, Clemson, SC 29634, USA; openape@g.clemson.edu (O.M.P.); cesarv@g.clemson.edu (C.V.); 2 School of Animal Sciences, Virginia Tech, Blacksburg, VA 24061, USA; gonf@vt.edu

**Keywords:** digestibility, summer annuals, legumes, grasses

## Abstract

**Simple Summary:**

A major challenge for livestock producers in a cool-season grass system is the seasonality of forage production, and in particular, the quantity and quality gap that usually occurs during the summer. Crabgrass is a warm-season annual crop with better forage quality than most other common summer annual grasses and can potentially be mixed with summer annual legumes. In this study, we evaluated on field plots the effect of intercropping two summer legumes (cowpea and lablab) with crabgrass on forage yield, nutritional composition, and fiber digestibility. We determined the degradability of the neutral detergent fiber under in vitro conditions using rumen fluid from lactating dairy cows. The results of this study showed that mixing crabgrass with cowpea and lablab partially mitigated the biomass yield drag from the legume monocultures while increasing crude protein concentration and fiber digestibility compared to the monoculture of crabgrass. Under the conditions of this study, the biggest impact of intercropping legumes was observed on the first of three harvests, which suggests that the evaluated legumes might not be an ideal complement for a multi-cut grass like crabgrass.

**Abstract:**

The objective of this study was to evaluate the effects of interseeding crabgrass (CG) with two annual summer legumes on forage nutritional composition, dry matter (DM) yield, and in vitro fiber digestibility. The study was conducted as a randomized complete block design with four replicates per treatment. Plots were randomly assigned to one of six forage mix treatments. Crabgrass, cowpea (CWP), and lablab (LL) were planted in monoculture or in mixtures, resulting in six treatments. Throughout the growing season (three cuts), CG had the highest biomass yield, followed by the CG grown in mixtures with CWP and LL, whereas the two annual legume monocultures had the lowest yield. Cowpea and LL planted in monocultures had the highest concentration of CP and fiber digestibility, while the CG monoculture had the lowest. Furthermore, growing CG in a mixture with CWP and LL boosted the CP concentration and fiber digestibility to intermediate levels to those observed between both legume monocultures and CG. Regardless of treatment, the highest forage quality and yield was observed in the first harvest, with a drastic decline in the following harvests. In conclusion, the benefits of mixing crabgrass with legumes might be less than expected and should be carefully evaluated by livestock producers, especially when considering the effects of DM yield, forage quality, and pasture seeding costs.

## 1. Introduction

The competitiveness of livestock producers depends in part on their ability to manage herds to reduce production costs while maintaining high levels of production and minimizing adverse environmental impact. Tall fescue [*Schedonorus arundinaceus* (Schreb.) Dumort] is the backbone of many cow–calf operations in the southeastern region of the US. One of the major challenges for producers in a cool-season grass system like tall fescue is the seasonality of forage production. Warm-season grasses and legumes (aka summer annuals) can complement perennial cool-season systems and extend grazing days and hay production during the summer when perennial grass production declines [1,2]. However, the high production costs of annual forages might impact profitability, therefore limiting their adoption in forage production systems [3]. Maximizing the forage yield and quality of summer annuals is a potential strategy that can increase farm returns by partially diluting the cost of establishment. 

Over the past few years, the interest in growing grasses in mixtures with legumes increased due to the potential benefits on soil fertility and forage quality. Legume species can fix N from the atmosphere [4] and can increase the crude protein (CP) concentration and the fiber digestibility of the forage when mixed with grasses [5], which may reduce the need for protein and energy supplements [6]. However, research on the potential trade-off between biomass yield and forage quality when different summer annual grasses and legumes are grown in monoculture or in mixes is scarce [7,8].

Crabgrass [*Digitaria sanguinalis* (L.) Scop.] is a drought-tolerant and warm-season annual crop with better forage quality than most other common summer annual grasses (e.g., pearl millet or sorghum–sudangrass hybrids) that can be planted in soils with a wide pH range [9] and support good animal performance for stocker calves and dairy cattle [10,11,12]. Furthermore, if properly managed, crabgrass can reseed itself from one year to another. In addition, growing crabgrass in mixtures with legumes could potentially increase residual soil N, improve forage yields of successive winter crops, and reduce fertilizer costs [13]. There are several summer annual legumes that could be beneficial to forage production systems in the southern part of the US. For example, cowpea [*Vigna unguiculata* (L.) Walp.] and lablab [*Lablab purpureus* (L.) Sweet] are vine-climbing legumes that are adapted to a wide range of soil pH, can tolerate some shade, and can produce high-quality forage [14,15,16]. 

We hypothesized that crabgrass mixed with summer annual legumes will increase forage quality, especially crude protein concentration and fiber digestibility, relative to crabgrass monoculture without affecting forage yield. Thus, the objective of this study was to evaluate the effect of intercropping cowpea and lablab with crabgrass on forage yield, nutritional composition, and fiber digestibility.

## 2. Materials and Methods

### 2.1. Experimental Sites and Climate Data

This study was conducted from April to September 2019 at the Simpson Research Farm, Clemson University, Pendleton, South Carolina (34°62′10.8″ N 82°73′31.5″ W). Soil is described as applying sandy loam with 2 to 6% slopes (ApB) and a land capability classification of IIe (web soil survey; www.nrcs.usda.gov, accessed on 18 August 2022). Weather and historic weather data (1981 to 2010) were collected from a weather station located in Sandy Springs, SC, using the National Centers for Environmental Information of the National Oceanic and Atmospheric Administration (NOAA, US Department of Commerce, www.noaa.gov, accessed on 18 August 2022). 

Rainfall amounts during the 2019 growing season were below the 30-year average during most of the growing season, with distinctly dry conditions experienced during May, July, August, and September (Table 1). Similarly, the recorded temperatures during the trial were, on average, higher than the 30-year mean.

### 2.2. Experimental Design

The trial was designed as a randomized complete block design with forage cut as a repeated measure. During the spring of 2019, the field was divided into four blocks, and within each of the blocks, one plot (1.5 m wide and 6.1 m long) was randomly assigned to one of six forage mix treatments. Crabgrass (CG; “Red river”), cowpea (CWP; “Iron and Clay”), and lablab (LL; “Ronagi”) were planted in monocultures (5.6, 56.1, and 33.6 kg/ha, respectively) or in mixtures of CG+CWP (2.8 + 28.0 kg/ha, respectively), CG+LL (2.8 + 16.8 kg/ha, respectively), or CG+CWP+LL (1.9 + 18.8 + 7.5 kg/ha, respectively). Plots were planted using a seven-row plot drill equipped with an Almaco cone. Fertilizer was applied to each plot before planting (22 kg N/ha, 56 kg P_2_O_5_/ha, and 45 kg K_2_O/ha) according to recommendations after soil analysis and after each harvest (23 kg N/ha). Plots were harvested when the crabgrass reached the late heading to early flowering stages of maturity (three harvests total).

### 2.3. Forage Processing and Analyses

The forage biomass of each plot was harvested three times (1 July, 31 July, and 13 September) using a Carter plot forage harvester (Carter Manufacturing Co., Brookston, IN, USA). After weighing the harvested biomass, samples from each plot were collected in plastic bags, immediately placed in a cooler with ice, and transferred to the laboratory for storage at −20 °C. Samples were thawed and dried at 55 °C in a forced-air oven for 48 h. The resulting dry matter (DM) concentration was used to determine DM yield (kg/ha). Dried samples were ground to pass through a 1 mm screen of a Wiley mill (Arthur H. Thomas, Philadelphia, PA, USA). Ground samples were dried at 105 °C for 24 h to determine analytical DM. Ash concentration was determined after combusting samples in a furnace for 3 h at 600 °C (Method 942.05, AOAC) [17]. For each sample, a subsample was separated and submitted to Cumberland Valley Analytical Services (Waynesboro, PA, USA) to determine the concentrations of N (Method 990.03, AOAC) [18] and water-soluble carbohydrates as described by Hall et al. [19]. Crude protein concentration was calculated as a percentage N × 6.25 after combustion analysis. Neutral detergent fiber (aNDFom) and acid detergent fiber (ADFom) concentrations were determined using an Ankom200 Fiber Analyzer (Ankom Technology, Fairport, NY, USA) and corrected for ash concentration. Sodium sulfite and α-amylase (Sigma no. A3306: Sigma Chemical Co., St. Louis, MO, USA) were included in the NDF analysis [20]. After determining the ADF, the fiber residue was incubated for 3 h in 72% sulfuric acid within 4 L jars that were placed in a Daisy II Incubator (Ankom Technology) for ADL determination.

Care and handling of animals used for collecting rumen contents and in situ incubations were conducted as outlined in the guidelines of the Clemson University Committee on Animal Use (AUP2022-0464). In vitro DM digestibility (IVDMD), in vitro true DM digestibility (IVTDMD), and in vitro NDF digestibility (IVNDFD) were determined using a Daisy II rotating jar in vitro incubator (Ankom Technology). Samples were incubated for 30 h following the procedures described by Ferreira and Mertens [21]. A composite inoculum was prepared with rumen fluid and solids collected before the morning feeding from two rumen-fistulated lactating dairy cows that were fed a diet containing 35.2% corn silage, 7.9% barley silage, 0.9% bermudagrass hay, and 56.0% concentrate mix (DM basis). To determine undegraded NDF (uNDF), a 0.25 g sample was weighed into F57 Ankom bags (Ankom Technologies) and incubated in the rumen of two rumen-fistulated and multiparous cows (one Jersey and one Holstein) for 240 h. The cows were fed the same diet described above. After the 240 h incubation, bags were weighed and subjected to aNDFom analysis as described previously. Harvested yield of potentially degradable NDF (pdNDF, kg/ha) was calculated by multiplying the concentration of the pdNDF by the DM yield of the corresponding plot.

### 2.4. Statistical Analysis 

Data were analyzed with the MIXED procedure of SAS (SAS version 9.4, SAS Institute Inc., Cary, NC, USA). The statistical model included the random effect of the block (df = 3), the fixed effect of treatment (df = 5), the interaction of block and treatment (df = 15), the fixed effect of harvest as a repeated measure (df = 2), the interaction between treatment and harvest (df = 10), and the random residual error (df = 36). The first-order autoregressive covariance structure was used to fit a time series-type covariance structure in which the correlation declines as a function of time. Significant differences and tendencies to differ were declared at *p* < 0.05 and *p* ≤ 0.10, respectively.

## 3. Results

### Forage Yield, Chemical Composition, and In Vitro Digestibility

**Figure 1:**
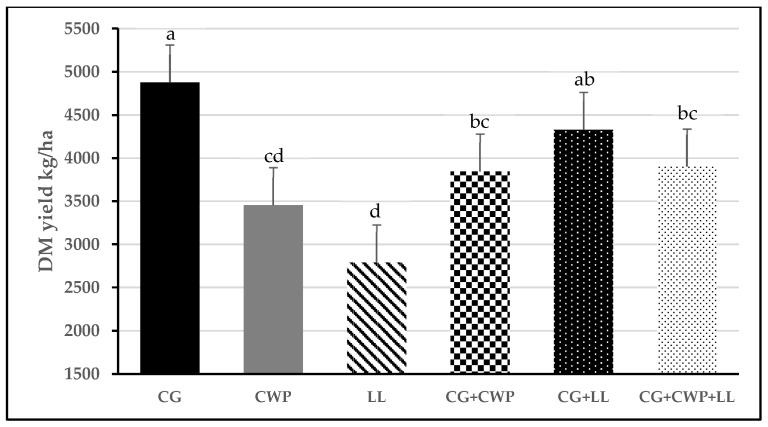
Biomass yield (kg DM per ha) of the different forage treatments. CG = crabgrass; CWP = cowpea; LL = lablab; CG+CWP = crabgrass + cowpea; CG+LL = crabgrass + lablab; CG+CWP+LL = crabgrass + cowpea + lablab. ^a–d^ Means with different letters differ (*p* ≤ 0.05). Vertical bars indicate standard errors of the mean.

Throughout the growing season, CG had the highest biomass yield, followed by the CG grown in mixtures with CWP and LL (mean = 4023 kg/ha), whereas the two annual legume monocultures had the lowest yield (3122 kg/ha, Figure 1). We observed a significant effect (*p* < 0.01) of harvest time on DM yield. Most of the harvested biomass was obtained in the first harvest (1908 kg/ha), followed by the second harvest (1605 kg/ha), and the lowest yield was observed in the last harvest of the growing season (893 kg/ha). 

**Figure 2:**
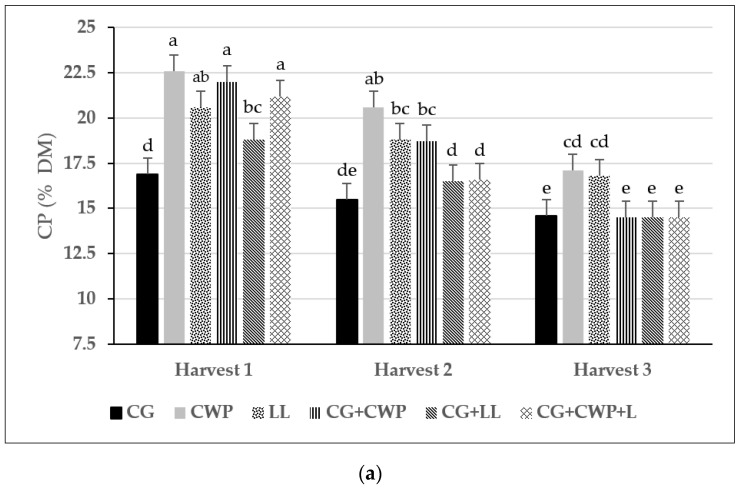

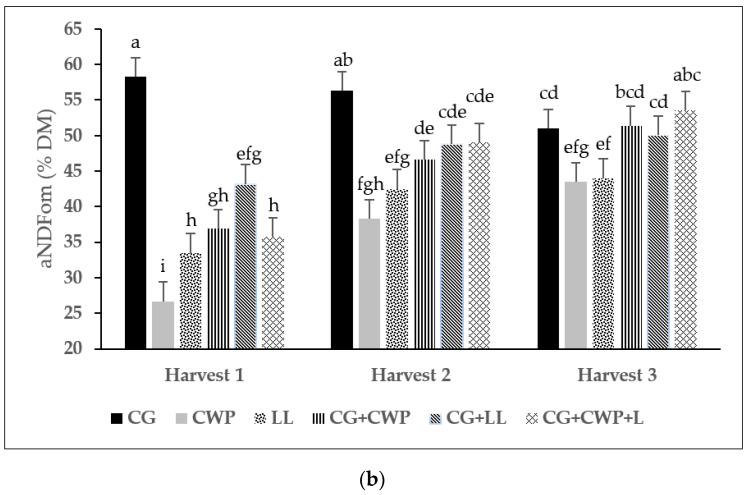
Interactions between forage treatments and harvests on CP (**a**) and aNDFom (**b**) concentrations (% DM). CG = crabgrass; CWP = cowpea; LL = lablab; CG+CWP = crabgrass + cowpea; CG+LL = crabgrass + lablab; CG+CWP+LL = crabgrass + cowpea + lablab. ^a–i^ Means with different letters differ (*p* ≤ 0.05). Vertical bars indicate standard errors of the mean.

The CWP had the lowest concentration of DM relative to all other treatments, which had similar DM concentrations (Table 2). Cowpea and LL planted in monocultures had the highest concentration of CP (20.1 and 18.7%, respectively), while the CG monoculture had the lowest (15.7%). Furthermore, growing CG in a mixture with CWP and LL increased the CP concentration of the forage to levels similar to those observed for the LL monoculture. However, we observed an interaction (*p* < 0.01) and a trend towards interaction (*p* = 0.08) between forage treatments and harvest time for DM and CP concentration, respectively. The forage treatment by harvest interaction reflected a larger increase in DM concentration between the first and third harvests for CG and all the mixes compared to monocultures of CWP and LL (Figure S1). On the contrary, CG had a lower CP concentration than the three forage mixes in the first harvest (16.9 vs. 20.7%) and second harvest (15.5% vs. 17.3%), but not in the last one (14.6 vs. 14.5%, Figure 2a). We also observed an interaction (*p* < 0.01) between forage treatment and harvests for aNDFom (Figure 2b). In the first harvest, the lowest aNFDom concentration was observed in both legume monocultures, while mixing grasses with the legumes resulted in intermediate levels of fiber concentration. In the second harvest, the CG still had the highest concentration of aNDFom, but the legumes in monoculture and the forage mixtures had similar aNDFom contents and were higher than the values observed in the first harvest. In the last harvest of the growing season, all treatments had similar aNDFom contents. The ADFom concentration followed a similar pattern to the aNDFom observed in the first harvest, and the treatment difference remained constant through the other two harvests. The concentrations of ADL (both on a DM and NDF basis) and WSC did not differ between forage treatments.

The concentration of uNDF on a DM basis differed among treatments but not on an aNDFom basis (Table 3). On a DM basis, CWP and LL had the lowest concentration of uNDF (9.3%); the three treatments with the grasses legumes mixtures were intermediate (10.8%), and the highest uNDF concentration was measured on the CG monoculture (12.5%). A similar pattern was observed for IVDMD and IVDMTD (Table 3). There was a treatment by harvest interaction for uNDF on an aNDFom basis (Figure S2). In the first harvest, CG and CG+LL had the lowest (15.3%); the CG+CWP+LL, CG+CWP, and LL treatments had an intermediate (17.2%); and CPW had the highest concentrations (25.6%) of uNDF (% of aNDFom). However, treatments did not differ on the second and third harvests.

**Figure 3:**
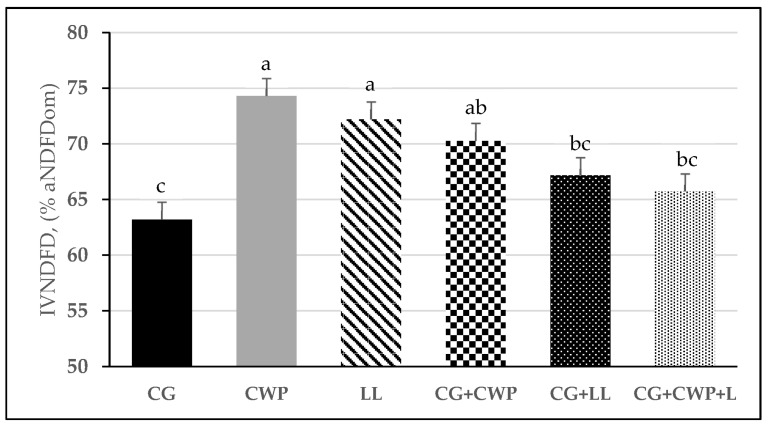
In vitro NDF digestibility (IVNDFD) of CG, CWP, and LL planted in monocultures or mixed. CG = crabgrass; CWP = cowpea; LL = lablab; CG+CWP = crabgrass + cowpea; CG+LL = crabgrass + lablab; CG+CWP+LL = crabgrass + cowpea + lablab. ^a–c^ Means with different letters differ (*p* ≤ 0.05). Vertical bars indicate standard errors of the mean.

Legume monocultures and CG+CWP had the highest IVNDFD, followed by CG+LL and CG+CWP+LL, while the lowest IVNDFD was observed in the CG treatment (Figure 3). 

The yield of pdNDF was highest for CG, but when CWP and LL were added to the grass, pdNDF yield was reduced by 36%. Furthermore, as a result of the higher DM yield and similar pdNDF concentrations (aNDFom-basis), CG pdNDF yield (kg/ha) was 63% higher compared to both legumes monocultures. 

**Figure 4:**
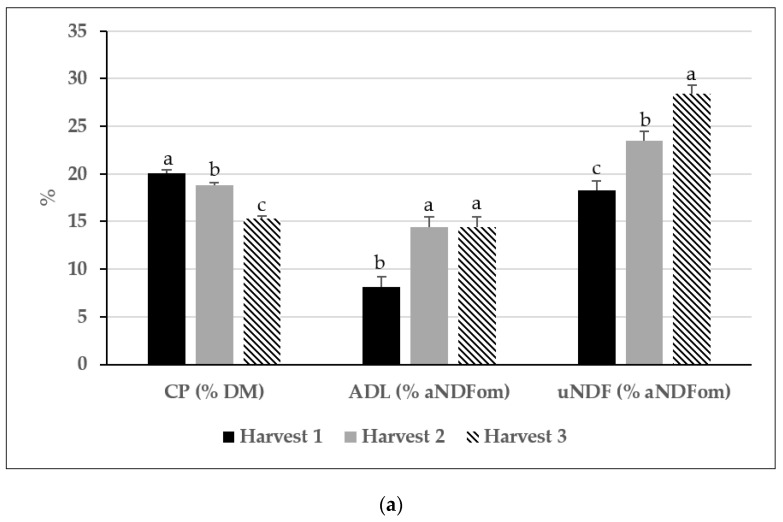

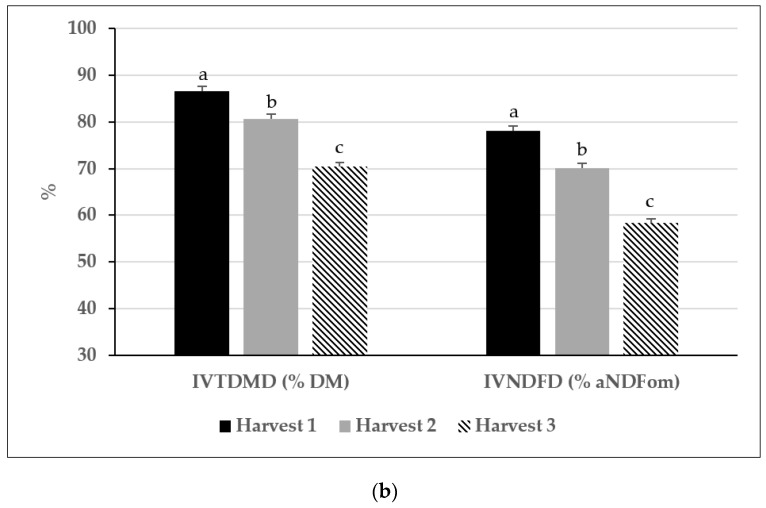
Concentration of forage CP (% DM), ADL (% aNDFom), and uNDF (% aNDFom) by harvest (**a**); in vitro true DM (IVTDMD) and aNDFom (IVNDFD) digestibility by harvest (**b**). ^a–c^ Means with different letters differ (*p* ≤ 0.05). Vertical bars indicate standard errors of the mean.

Other chemical components and the in vitro DM and fiber digestibility were significantly affected by harvest time. For example, CP concentration consistently decreased between the first and last harvests, while ADL (% aNDFom) and uNDF (%aNDFom) followed the opposite pattern (Figure 4a). Additionally, the first harvest had the highest (86.6 and 78.0%), the second harvest had an intermediate (80.6 and 70.1%), and the last harvest had the lowest (70.1 and 58.3%) IVTDMD and IVNDFD, respectively (Figure 4b).

## 4. Discussion

### Forage Yield, Chemical Composition, and In Vitro Digestibility

Under the conditions of this study, the forage DM yield was reduced by 21.2, 14.3, and 25.5% when CG was grown in combination with CWP, LL, or CWP + LL, respectively. Bryan and Materu did not observe an impact on the forage DM yield when interseeded cowpea and corn were compared with a corn monoculture [8]. Similarly, Armstrong et al. reported no differences in DM yields when corn was grown alone or mixed with lablab or velvet bean [6]. On the contrary, and in agreement with our observations, Oskey et al. observed that mixing pearl millet with CWP reduced the DM yield by 8.3% [7]. Both legumes evaluated in this study had a lower DM content compared to CG (Table 2), and with a lower plant population of the grass in the forage mix compared to the monoculture treatment, the overall DM yield per ha was likely penalized. Furthermore, CWP and LL have a slow regrowth after grazing or harvest, which could further impact the yield potential of the forage mixes after the first harvest [22]. In addition, the poor regrowth of the legumes can also result in empty spaces in the field that would not produce forage or could be filled by undesired weeds. Therefore, the results of the current and previous studies [7,22] provide further evidence that annual summer legumes such as cowpea and lablab may be a better option for single-cut forage mixes such as sorghum or corn. Finally, drought conditions during the growing season might have further penalized cowpea and lablab regrowth potential.

Chemical compositions of CG, CWP, and LL observed in this trial were within the reported ranges for these annual forages [11,23,24]. The results of this study demonstrate that adding CWP alone or combined with LL to CG can increase the CP content of the forage mix compared to the CG monoculture (Table 2). The 17.4 and 11.4% increase in CP concentration observed between CG and CG+CWP and CG+CWP+LL, respectively, is similar to the 13 to 23% increase in CP reported in several studies when intercropping corn with several annual legumes [6,25,26]. Furthermore, Brown et al. and Lauriault and Kirksey reported a 16 to 38% increase in CP in winter annual grasses and legumes [5,27]. Oskey et al. observed a significant but lower (7.4%) increase in CP concentration when pearl millet was intercropped with cowpea [7]. In addition, Angadi et al. [28] observed a numerical increase (6.4%) in CP concentration when cowpea and lablab were intercropped with forage sorghum. While the increase in CP content observed in this study may have a minor impact on animal performance, it may help reduce the cost of protein supplementation in the diet. However, producers would probably need to increase the amount of land area to compensate for the decrease in DM yields (Figure 1), with a concomitant increase in the production cost.

In line with the observations of Contreras-Govea et al. [29], fiber concentration was higher in the grass monoculture than in the legumes or the forage mixes. Grasses usually contain higher fiber concentrations than legumes [30]. Therefore, growing grasses in mixtures with legumes containing lower fiber concentrations likely reduces the NDF concentrations in the harvested forage [5]. However, Oskey et al. [7] reported no difference when pearl millet was intercropped with cowpea. It is possible that the proportion of the annual legume in the mixture was not large enough to have a significant impact on the fiber content of the forage mix [29]. The lower contribution of cowpea to the mix might also explain the smaller impact on CP concentration reported by Oskey et al. [7] and Angadi et al. [28] compared to the current study. Similarly, Contreras-Govea et al. [29] reported the greatest impact on forage nutrient composition when the contribution of lablab to the mixture increased as corn planting density decreased. The interactions observed between treatments and harvest for CP (tendency to difference) and aNDFom suggest that the plant composition of legume-containing plots might have changed during the growing season. For example, the difference in CP concentration between CG and the three forage mix CWP declined from 3.8% units in the first harvest to no difference in the third harvest. Moreover, the difference in aNDFom concentration follows a similar pattern, and there was no difference in fiber content between CG and the forage mixes in the third harvest (51.0 vs. 51.7%). These results, in addition to the visual assessment of the plots, suggest that after the first harvest, legumes grew back at slower rates than the CG and were likely outcompeted by CG grass (established in the mixes or volunteer) or weeds. Furthermore, the drought experienced during the last two harvests might have exacerbated the regrowth disadvantage of the legumes compared with the CG.

We observed a 12 and 16% increase in IVTDMD and IVNDFD, respectively, between CG and the two legume monocultures. Similarly, as reported in the current study, La Guardia Nave and Corbin [29] reported a 23% increase in IVTDMD between CG and CWP in a two-year study. In addition, growing CG in mixtures with one or both legumes resulted in an overall improvement in fiber digestibility compared to the grass monoculture, in particular, the CG+CWP mix (Figure 3). In cool-season legumes, fiber digestibility is usually lower, and ADL is higher than in grasses [5,31], which is not consistent with the results observed in this current study. Although not significant, the legume monocultures and the forage mixes had a higher ADL concentration on an aNDFom-basis than the CG monoculture, which was not reflected on the IVNDFD. Further studies are warranted to further evaluate ruminal fiber degradation kinetics within and among summer annual legumes.

Under the conditions of this study, the consistent decrease in forage yield and quality observed between the first, second, and third harvests suggests that, regardless of forage treatment, maximum animal production potential will likely be achieved only in the earlier harvest. Furthermore, the combined low yield and forage quality observed in the third harvest suggest this last harvest might be avoided.

## 5. Conclusions

As hypothesized, seeding legumes with crabgrass improved the quality of the forage relative to that of crabgrass alone, although this improvement occurred at the early cuts and not so much at the late cuts. Additionally, cowpea seemed to have a more consistent effect than lablab on forage quality. In regard to yield, seeding legumes with crabgrass did not increase DM yield. Even more, DM yields decreased when crabgrass was mixed with some legumes. In conclusion, the benefits of mixing crabgrass with legumes might be less than expected and should be carefully evaluated by livestock producers, especially when considering the effects of DM yield, forage quality, and pasture seeding costs.

## Figures and Tables

**Table 1:** Total monthly precipitation (mm), mean monthly temperature (°C), and 30-year historical average for Sandy Springs, SC, during the 2019 growing season ^a^.

Month	Precipitation	30-Year Avg.	Temperature	30-Year Avg.
April	73.9	91.7	19.3	15.1
May	34.8	96.5	25.8	19.3
June	103.6	122.7	26.9	24.1
July	8.5	115.8	29.9	25.8
August	59.9	114.8	29.1	25.3
September	9.9	106.7	28.2	21.7

^a^ Data obtained from NOAA, US Department of Commerce (https://www.nrcs.usda.gov, accessed on 18 August 2022).

**Table 2:** Nutritional quality of CG, CWP, and LL grown in monocultures or in mixtures ^1^.

Item	CG	CWP	LL	CG+CWP	CG+LL	CG+CP+LL	SEM	*p*-Value
DM, %	31.8 ^ab^	26.5 ^c^	31.3 ^ab^	28.8 ^bc^	32.7 ^ab^	30.3 ^ab^	1.36	0.03
Ash, % DM	13.6 ^c^	16.6 ^a^	15.4 ^ab^	14.6 ^bc^	16.0 ^a^	14.0 ^bc^	0.70	0.01
CP, % DM	15.7 ^d^	20.1 ^a^	18.7 ^ab^	18.4 ^b^	16.6 ^cd^	17.4 ^bc^	0.49	<0.01
aNDFom, % DM	55.2 ^a^	36.2 ^d^	40.0 ^cd^	45.0 ^bc^	47.4 ^b^	46.1 ^b^	1.92	<0.01
ADFom, % DM	32.0 ^a^	24.0 ^c^	25.7 ^c^	28.4 ^bc^	29.6 ^ab^	28.7 ^b^	1.07	<0.01
ADL, % DM	5.00	5.41	5.35	5.99	5.61	5.15	0.59	0.73
ADL, % aNDFom	9.3	14.8	13.6	13.5	11.6	10.9	1.56	0.21
WSC, % DM	7.4	8.6	7.9	7.2	7.2	7.6	0.38	0.11

^1^ CG = crabgrass; CWP = cowpea; LL = lablab; CG+CWP = crabgrass + cowpea; CG+LL = crabgrass + lablab; CG+CWP+LL = crabgrass + cowpea + lablab. Means with different superscripts in the same row differ (*p* < 0.05).

**Table 3:** Effect of forage treatments on undigestible NDF concentration and in vitro DM digestibility ^1^.

Item	CG	CWP	LL	CG+CWP	CG+LL	CG+CP+LL	SEM	*p*-Value
uNDF_240_, % DM ^2^	12.5 ^a^	9.3 ^bc^	9.0 ^c^	10.4 ^b^	11.5 ^ab^	10.6 ^b^	0.56	<0.01
uNDF_240_, % aNDFom ^2^	23.1	25.5	22.3	22.8	24.1	22.6	1.26	0.40
pdNDF, % aNDFom ^3^	76.9	74.5	77.7	77.2	75.9	77.4	1.26	0.40
IVDMD, % DM ^4^	65.8 ^d^	74.3 ^a^	73.1 ^ab^	71.3 ^b^	68.1 ^cd^	69.9 ^bc^	1.29	<0.01
IVTDMD, %DM ^5^	75.5 ^c^	82.5 ^a^	81.2 ^a^	79.9 ^ab^	77.4 ^bc^	78.7 ^ab^	1.41	0.01
pdNDF, kg DM/ha	2183 ^a^	804 ^c^	804 ^c^	1300 ^b^	1585 ^b^	1342 ^b^	212	<0.01

^1^ CG = crabgrass; CWP = cowpea; LL = lablab; CG+CWP = crabgrass + cowpea; CG+LL = crabgrass + lablab; CG+CWP+LL = crabgrass + cowpea + lablab. ^2^ uNDF240 = undegraded neutral detergent fiber (after 240 h of fermentation). ^3^ pdNDF = potentially degradable neutral detergent fiber. ^4^ IVDMD = in vitro 30 h dry matter digestibility. ^5^ IVTDMD = in vitro 30 h true dry matter digestibility. Means with different superscripts in the same row differ (*p* < 0.05).

## Data Availability

Not applicable.

## Supplementary Materials

The following supporting information can be downloaded at: https://www.mdpi.com/article/10.3390/ani13142305/s1, Figure S1: Interactions between forage treatments and harvest on DM concentration (%); Figure S2: Interactions between forage treatments and harvest on uNDFom concentration (% of aNDFom).
