# Supplementary material for: Nutritional Composition and In Vitro Ruminal Digestibility of Crabgrass (Digitaria sanguinalis (L.) Scop.) in Monoculture or Interseeded with Cowpea (Vigna unguiculata (L.) Walp) and Lablab (Lablab purpureus (L.) Sweet)
Source: Animals (Basel). 2023 Jul 14;13(14):2305. doi: 10.3390/ani13142305 (PMC10376878; doi:10.3390/ani13142305)
Supplement: Supplementary file 1 [file animals-13-02305-s001.zip › animals-2446157-supplementary.pdf]

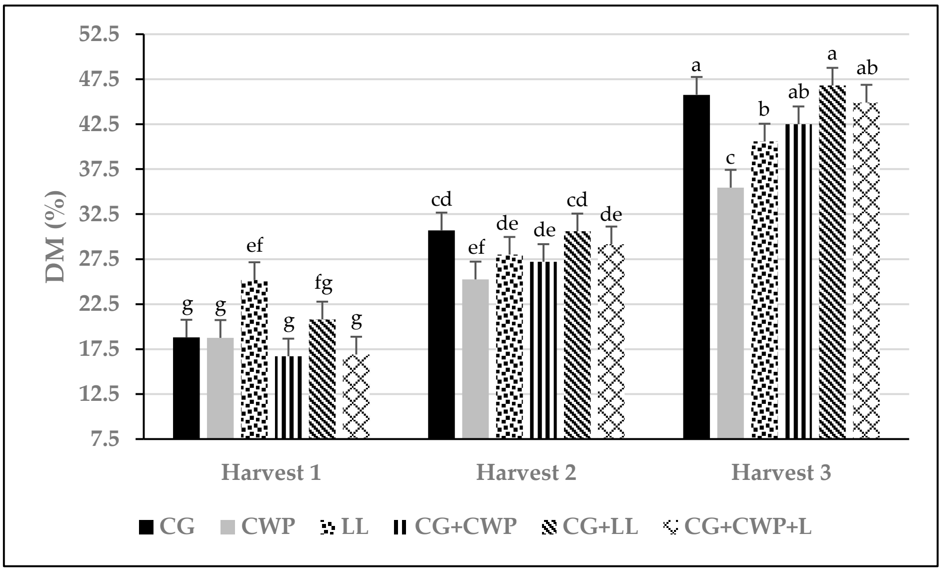


**Figure S1**. Interactions between forage treatments and harvest on DM concentration (%). CG= Crabgrass; CWP= cowpea; LL = lablab; CG+CWP = crabgrass + cowpea; CG+LL = crabgrass + lablab; CG+CWP+LL = crabgrass + cowpea + lablab. [a–g]Means with different letters differ ($p \leq 0.05$). Vertical bars indicate standard errors of the mean.

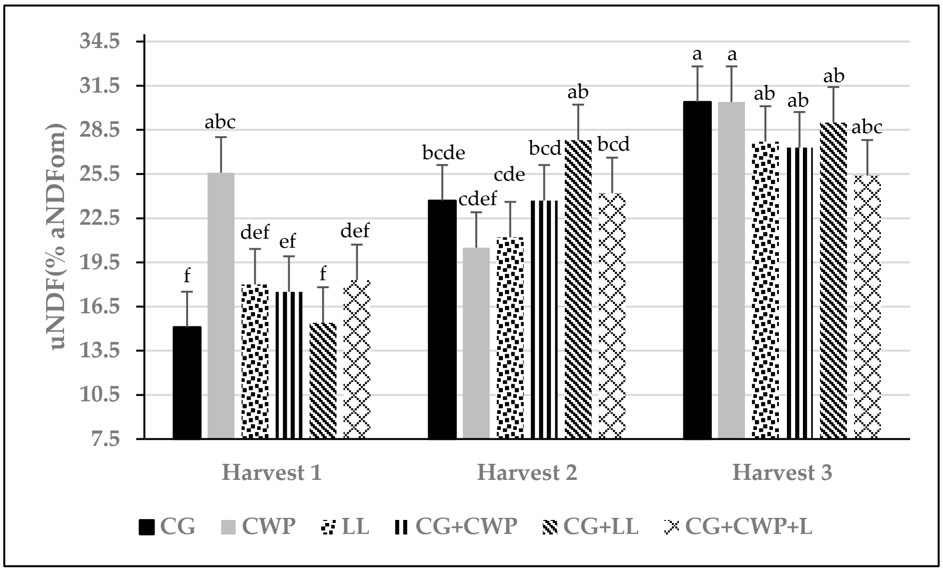


**Figure S2**. Interactions between forage treatments and harvest on uNDF concentration (% of aNDFom). CG= Crabgrass; CWP= cowpea; LL = lablab; CG+CWP = crabgrass + cowpea; CG+LL = crabgrass + lablab; CG+CWP+LL = crabgrass + cowpea + lablab. [a–f]Means with different letters differ ($p \leq 0.05$). Vertical bars indicate standard errors of the mean.
